# Short-term clinical effect of 3D printing techniques on the correction of complex malformations

**DOI:** 10.1007/s00132-019-03717-6

**Published:** 2019-05-22

**Authors:** Jieyu Liang, Hongbin Guo, Jingyuan Guo, Juyu Tan, Yihe Hu, Kanghua Li, Min Wang

**Affiliations:** 1 Department of Orthopedics, Xiangya Hospital, Central South University, 410008 Changsha, Hunan China; 2 Department of Orthopedics, the third Hospital of HuaiHua City, 418000 Huaihua, Hunan China; 3 Department of EndocrineXiangya Hospital, Central South University, 410008 Changsha, Hunan China

**Keywords:** Computed tomography, Disability evaluation, Osteotomy, Orthopedics, Patient outcome assessment, Computertomographie, Evaluation der Körperbehinderung, Osteotomie, Orthopädie, Bewertung der Patientenergebnisse

## Abstract

**Background:**

To explore the clinical effects of 3D printing techniques on the correction of complex malformation.

**Method:**

A computed tomography (CT) scan was used to collect data on malformations of patients and the orthopedic plan was made by virtual manipulation of the reality before surgery. The results of the virtual orthopedics were compared with the expected results. A guide plate for osteotomy was also utilized when necessary. The actual operation was carried out according to the plan.

**Results:**

The average age of the 11 patients was 19.09 years (19.09 ± 6.93 years) and the average follow-up was 16 months (16 ± 15.11 months). The symptoms were obviously improved. The preoperative World Health Organization Disability Assessment Schedule (WHODAS 2.0) score, modified Barthel index and Functional Independence Measure (FIM) score in patients were 70.45 ± 15.75, 96.55 ± 3.78 and 121.36 ± 4.15, respectively and correspondingly 53 ± 12.75, 98.82 ± 1.66 and 123.82 ± 4.60 after surgery, respectively. There were significant differences before and after surgery (*P* < 0.05).

**Conclusion:**

The use of 3D printing technology can provide intuitive and accurate help for the correction of complex limb malformations and greatly facilitates the communication between doctors and patients. The FIM score is suitable for the evaluation of the curative effect before and after the treatment of patients with complex malformations.

## Zusammenfassung

**Hintergrund:**

Der klinische Effekt von 3‑D-Drucktechniken zur Korrektur komplexer Fehlbildungen soll untersucht werden.

**Methode:**

Eine Computertomographie (CT) wurde zur Erfassung von Daten über Fehlbildungen bei Patienten verwendet, und die orthopädische Planung wurde vor der Operation durch eine virtuelle Manipulation der Realität erstellt. Die Ergebnisse der virtuellen orthopädischen Daten wurden mit den erwarteten Ergebnissen verglichen. Eine Führungsplatte für die Osteotomie wurde, sofern erforderlich, ebenfalls verwendet. Die Operation wurde plangemäß durchgeführt.

**Ergebnisse:**

Das Durchschnittsalter der 11 Patienten lag bei 19,09 Jahren (19,09 ± 6,93 Jahre), und der durchschnittliche Follow-up betrug 6 Monate (16 ± 15,11 Monate). Die Symptome verbesserten sich deutlich. Der präoperative Score des World Health Organization Disability Assessment Schedule (WHODAS 2.0), der modifizierte Barthel-Index und der Functional-Independence-Measure(FIM)-Score der Patienten betrugen 70,45 ± 15,75, 96,55 ± 3,78 bzw. 121,36 ± 4,15 sowie nach der Operation 53 ± 12,75, 98,82 ± 1,66 bzw. 123,82 ± 4,60. Es gab signifikante Unterschiede vor und nach der Operation (*p* < 0,05).

**Schlussfolgerung:**

Die Verwendung der 3‑D-Drucktechnik dient als unmittelbare und präzise Hilfe bei der Korrektur komplexer Extremitätenfehlbildungen und erleichtert die Kommunikation zwischen Arzt und Patient deutlich. Der FIM-Score ist zur Bewertung des Heilungseffekts vor und nach der Behandlung der Patienten mit komplexen Fehlbildungen geeignet.

## Background

The 3D printing technology appeared in the mid-1990s. The printer is equipped with printing materials such as liquid or powder. After being connected to a computer, the printing material is stacked up by computer control, and finally the blueprint on the computer is turned into real objects. This printing technology is called 3D stereoscopic printing technology. The medical application of 3D printing is rapidly expanding and it is expected to make a fundamental change to the traditional medical healthcare model [1]. In the actual clinical practice, CT and MRI scans are used to scan the affected side of patients, and data can be imported into Mimics3D (Mimics 3D Software, Materialise Company, Leuven, Belgium) software to generate virtual realistic parts of patients, and eventually resulting in the printing of finished products. An intraoperative osteotomy guide plate can be prepared during the same period so as to help clinicians complete the actual precise operation. The work of 3D printing technology in this department is reported.

## Methods

### General information

From 2014 to 2017, there were 11 patients incorporated in the study, including 5 males and 6 females, with an average age of 19.09 years (19.09 ± 6.93 years). The follow-up time lasted for 2–56 months with an average duration of 16 months (16 ± 15.11 months). There were 2 cases with an affected femur, 2 with a foot, 4 with a tibia, 1 with an upper arm, 1 with a forearm, and 1 with a femur combined with a tibia. Furthermore, there were 5 cases with sequelae of trauma, 3 cases with congenital malformations and 3 cases of bone disease (tumor, osteomyelitis, and rickets, respectively). In addition, 2 patients underwent internal fixation, 3 underwent external fixation using Taylor spatial frames and the remaining 6 patients were treated using Ilizarov spatial frames.

### Preoperative preparation

All patients were documented using conventional static photography of the appearance and dynamic photography of the function before the operation. According to the Paley principles [2], the center of rotation of angulation (CORA) was determined on the X‑ray by marking lines along the anatomic axis and the angle and the specific location including the distance from bony landmarks of the osteotomy were accurately calculated.

### 3D printing

The malformation of patients was scanned by CT and relevant data were collected for 3D printing, with a scanning thickness of 0.5 mm. Data were exported and stored in a DICOM format. A DICOM file extraction was achieved by using the Mimics 10.0 software. The reverse model was obtained by reverse engineering technology, meanwhile, it should be ensured that the guide plate designed through computer programming could be accurately attached on the surface of bone. Subsequently, STL files generated by the bone and guide plate were imported into the 3D printer, followed by the performance of 3D printing. Virtual manipulation of the reality was carried out on the computer, associated with the verification of the preoperative plan and postoperative results. All the printed materials used in this study were ABS resin. After the printing was completed, the guide plate and bone model were check and confirmed and the guide plate was sent to the operation room for disinfection and reservation.

### Operative method

**Fig. 1:**
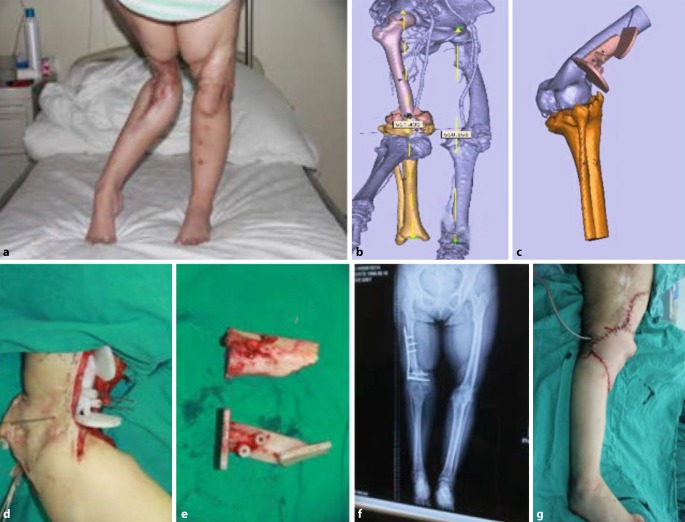
3D guide plate assisted acute osteotomy and free flap transplantation with internal fixation. A female patient, aged 26 years old, had malformation of lower limbs after trauma for more than 20 years. Left lower extremity had been treated in the external hospital with malformation correction and arthrodesis. However, there were still residual genu recurvatum of the right lower extremity, excessive length, knee hyperextension and sticking scar. **a** Preoperative appearance. **b** Preoperative virtual manipulation. **c** Intraoperative guide plate assisted osteotomy. **d** Guide plate and cut bone. **e** Postoperative imaging. **f** Postoperative appearance

**Fig. 2:**
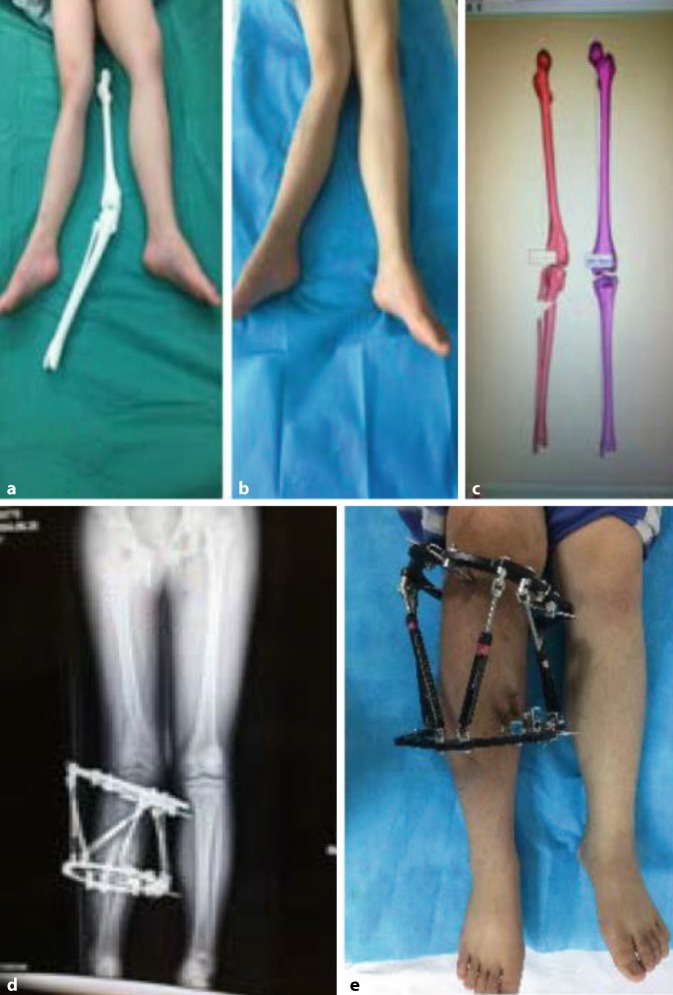
A male pediatric patient, aged 12 years old, had external rotation 15°, eversion 25° and shortening 3 cm malformation of the right lower extremity induced by systemic multiple non-ossifying fibroma involving the epiphysis. The patient underwent osteotomy of the upper segment of the right tibia and fibula, and correction with Taylor spatial frame. **a** Preoperative 3D printing. **b** Preoperative appearance. **c** Preoperative virtual manipulation. **d** Postoperative X‑ray imaging. **e** Postoperative orthopedic effect

Following general anesthesia and general disinfection the operation was performed in strict accordance with the preoperative 3D printing plan. Some of the complex osteotomies were assisted by a guide plate during the operation. Internal fixation or external fixation was used in time after the completion of osteotomy. The external fixator was gradually corrected 7 days after the operation, 0.75 mm/day in adults and 1 mm/day in children step by step on the basis of the preset plan (Figs. 1 and 2).

### Postoperative care

The monthly outpatient review was conducted for real-time adjustment. Furthermore, the overall time with the fixator for adults was three times as long as the duration of the adjustment, and two times for children. It was also necessary to refer to the callus density provided by X‑ray when removing the fixator, and the brace should be worn for 2–3 months after the removal.

### Observational indexes

All patients underwent an X‑ray review, as well as appearance and function photography by regular follow-up visits. Corresponding results were compared with those before the operation. With the assistance of an assistant, the patients and their families were required to fill in forms of WHODAS 2.0 score, the modified Barthel index and FIM scores on all items to record the preoperative and postoperative scores of each patient.

### Statistical analysis

The SPSS 20 software was used for statistical analysis, and all the data were tested in normality. Measurement data were expressed as mean ± standard deviation (x ± SD). The significance level was *P* = 0.05. All the data in WHODAS II group were in normal distribution, and the self-control t‑test was used. The data of the modified Barthel index and FIM groups did not conform to the normal distribution, and the non-parametric rank sum test was utilized accordingly. Besides, correlation of patients in the modified Barthel and FIM groups was achieved using Wilcoxon sign rank test before and after the operation.

## Results

All the patients effectively completed the forms. The results of the WHODAS II, modified Barthel index and FIM scores were as follows before and after the operation. There were significant differences before and after the operation (*P* < 0.05).

### General appearance and functional recovery

The appearance and function of all patients were improved to varying degrees at the last follow-up. Without the support of an assistive device, all patients were able to perform most or all of their daily activities independently.

### Statistical results of three scoring systems

The results of the preoperative and postoperative score systems are given in Table 1.

**Table 1:** Results of the three scoring systems for the 11 patients (vertical columns) in this study

Separate statistical results of three scoring (S) systems to 11 patients (P)											
	P1	P2	P3	P4	P5	P6	P7	P8	P9	P10	P11
*WHO-DAS II*											
Preoperative S	95	83	69	53	59	91	48	82	63	74	58
Postoperative S	78	58	43	40	40	69	43	44	53	63	52
Results	Preoperative 70.45 ± 15.75; Postoperative 53 ± 12.75; t = 5.852; *P* = 0.00										
*Modified Barthel index*											
Preoperative S	86	96	96	98	100	98	100	98	97	96	100
Postoperative S	97	100	100	100	100	99	100	100	97	96	100
Results	Preoperative 96.82 ± 3.92; Postoperative 99 ± 1.55; z = −2.214; *P* = 0.027										
*FIM*											
Preoperative S	120	122	123	122	124	110	125	123	123	119	124
Postoperative S	125	126	125	126	126	111	126	126	125	120	126
Results	Preoperative 121.36 ± 4.15; Postoperative 123.82 ± 4.60; z = 2.956; *P* = 0.03										

The preoperative score of WHO-DAS II, modified Barthel and FIM in included patients was (70.45±15.75), (96.55±3.78) and (121.36±4.15), respectively. Corresponding score was (53±12.75), (98.82±1.66) and (123.82±4.60) after operation, respectively. There were significant differences between groups before and after operation (*P*<0.05)

*P1–11* patients 1–11, *S* Scoring System, *WHO-DAS II* World Health Organization Disability Assessment Schedule, *FIM* functional independence measure

The preoperative scores of WHO-DAS II, modified Barthel index and FIM in the patients were 70.45 ± 15.75, 96.55 ± 3.78 and 121.36 ± 4.15, respectively. Corresponding 53 ± 12.75, 98.82 ± 1.66 and 123.82 ± 4.60 after operation, respectively. There were significant differences between groups before and after operation (*P* < 0.05).

### Correlation analysis between FIM score and modified Barthel index

The results of the correlation analysis of the two scores are given in Table 2.

**Table 2:** Correlation results for preoperative and postoperative data of the FIM and modified Barthel index

Correlation analysis between FIM and modified Barthel index				
*Statistical results of correlation between FIM and modified Barthel index before the operation*				
			VAR00001	VAR00002
Spearman’s rho test	VAR00001	Correlation coefficient	1.000	0.664^a^
		Sig. (bilateral)	–	0.026
		*N*	11	11
	VAR00002	Correlation coefficient	0.664^a^	1.000
		Sig. (bilateral)	0.026	–
		*N*	11	11
*Statistical results of correlation between FIM and modified Barthel index after the operation*				
			VAR00001	VAR00002
Spearman’s rho test	VAR00001	Correlation coefficient	1.000	0.815^b^
		Sig. (bilateral)	–	0.002
		*N*	11	11
	VAR00002	Correlation coefficient	0.815^b^	1.000
		Sig. (bilateral)	0.002	–
		*N*	11	11

^a^The correlation was significant when the confidence level (bilateral) was 0.05

^b^The correlation was significant when the confidence level (bilateral) was 0.01

*VAR00001* Preoperative FIM score group, *VAR00002* Preoperative improved Barthel group

The preoperative and postoperative data of the two scales were often highly correlated.

## Discussion

The application of 3D printing can provide many benefits in the field of medicine, including customizing personalized medical products, medicines and equipment with cost-effectiveness and high efficiency [3–7]. The implementation of this technology has also brought great help to patients and doctors [8]. Correction of bone malformations has been a major problem for doctors. In the past, such problems could only be solved through repeated preoperative comparisons. The actual process of the operation is counterintuitive with large errors. There was large range of tissue exfoliation, a great amount of bleeding, the number of X-ray fluoroscopy during the operation was increased, which was disadvantageous to doctors and patients, poor accuracy and long operation duration, and the clinical effect of operation varies with the experience of the different doctors. In this respect, before the development of 3D printing, there was no simple, quick and accurate method to guide the implementation of osteotomy. The bone model designed by 3D printing technology is practical, and the guide plate is exactly matched with the bone. Doctors can complete osteotomy simulation in the printed bone via a guide plate before an operation and can repeat the same operation in the diseased bone of patients intraoperatively [9, 10].

The 3D printing technique is a typical combination of engineering and medicine in the treatment of complex malformations in China. In this process, doctors are always in the dominant position, and engineers cannot be excessively relied upon since 3D printing technique is only a means of implementing the medical process. Furthermore, the correction of complex malformations requires clinician to have extensive professional experience and solid theoretical basis, not only depending on the assessment with the naked eye. Especially for those patients with multiple vertex malformations, obvious deformity vertex should be identified, associated with the identification of other hidden deformity vertexes through precise marking before surgery, thereby achieving complete correction. Preoperative marking was carried out in all cases in this study in strict accordance with the Paley principles of deformity correct [1] to determine the number of deformity vertexes and the actual angle to be corrected. Subsequently, the requirements and various data are delivered to the engineer of the 3D printing technique to produce the sample and the guide plate of osteotomy for simulation and practical operation as required. In this approach, it greatly improves the accuracy of orthopedic results [11].

The authors have some experience and understanding with respect to the determination of the type of fixation used after osteotomy. Firstly, gradual correction should be avoided if acute osteotomy is available, unless patients need gradual postoperative correction such as lengthening. Secondly, the external fixation should be avoided if internal fixation is possible. The internal fixation is more comfortable with reliable fixation and convenient nursing. Thirdly, the Taylor frame and Ilizarov external frame are commonly used in external frame fixation. The former is obviously better than the latter in the aspect of biomechanical stability and adjustment accuracy [12–14], and its ability to correct rotation is especially prominent; however, the disadvantage is that the costs and components replacement of Taylor frame in the later stage is relatively high, and it requires regulation prescription provided by both the doctor and the engineer, so it was only applied in patients with complex malformations in this study. In addition, the external fixator has advantages that internal fixation does not possess, such as slow adjustment after surgery, and the rare chance of neurovascular injury, etc. For the daily extension of the outer frame, corresponding adjustment should be made according to the callus density shown on the X‑ray during each re-examination. Instead of simple adjustment at a speed of 1 mm/day mentioned in the textbook, adjusting the speed of 0.75 mm/day in adults and 1 mm/day in children was more suitable in our experience.

A more general international rating scale should be selected for the evaluation of results of malformation correction. The WHO-DAS II score is an internationally recognized scale for evaluating the disability of the limb, which is convenient to be completed in a limited period of time; however, it is biased in the field of mental and cognitive changes, and it is not appropriate for children regarding the item of sexual life, housework and other projects. The modified Barthel index and FIM motor scale have become the most widely used daily activity assessment scales in clinical application. Besides, in previous literature, the results of the two scales were often highly correlated. It was believed in the current study that the modified Barthel index reflected the improvement of daily activity in the observed patients, but not including the improvement in the cognitive ability before and after treatment. More importantly, patients with correction of complex malformations were usually unable to improve their ability in the short term. Nevertheless, with the gradual improvement of the appearance and function of malformation, the ability of self-cognition could certainly be improved. For the convenience of comparison, the data of FIM were collected only 3 days after admission and during the recent follow-up.

There are also some shortcomings of the 3D printing technique in the implementation of complex malformation correction. For example, image processing and 3D printing takes a long time, which cannot be used for emergency treatment. Many hospitals are in a poor condition and do not have relevant technology and equipment and it is not conducive to the extensive development in those hospitals. In addition, the guide plate cannot be sterilized by high temperature due to material defects. Meanwhile, the current application rate is low in China, and there is the restriction of ethical issues; however, these situations do not stop the trend of 3D printing becoming more and more important in the biomedical field, and the only thing required is verification over time [4]. The sample size in this study was relatively small and the duration of follow-up was not long enough, which will be critical in the future.

To sum up, 3D printing technology can provide intuitive and accurate help for the correction of complex limb malformations, and greatly facilitates the communication between doctors and patients, and it is worth popularizing. The FIM score is suitable for the overall assessment of the curative effect before and after the treatment of patients with complex malformations.

## Abbreviations

ABS: Acrylonitrile butadiene styrene plastic
CORA: Center of rotation of angulation
CT: Computed tomography
3D: 3-dimensional
DICOM: Digital imaging and communications in medicine
FIM: Functional independence measure
Mimics: Materialise’s interactive medical image control system
MRI: Magnetic resonance imaging
SPSS: Statistical Package for the Social Sciences
STL: Stereolithography
WHO-DASII: World Health Organization Disability Assessment Schedule 2.0
